# And Yet Another Journal

**Published:** 1886-12

**Authors:** 


### AND YET ANOTHER JOURNAL.

Last month we printed the announcement of *The Western Dental Journal,* to be published in Kansas City. Since then we have received the prospectus of *The Dental Review,* a monthly journal to be published in Chicago. It is an encouraging indication when first-class professional magazines multiply, for it shows that due and proper attention is being paid to that literature without which there can be no organized profession.

The prospectus of the new journal gives no hint as to who are to be its editors, but if it was desired that it should be conducted in an entirely impersonal manner the secret has not been well kept, for uncontradicted rumor organizes the staff as follows:

Editor in Chief, Dr. A. W. Harlan.

Associate Editors, Dr. J. G. Reid, Dr. J. W. Wassall, Dr. Louis Ottofy and Dr. Davis.

This is a strong corps, and one, we doubt not, that will be entirely competent to wipe out the stain which, the prospectus intimates, rests upon our professional periodical literature—"a deplorable lack" of editorial ability, or of editorial energy and wisdom. It is promised that the present editorial inefficiency of our journalism will, in some measure at least, be corrected in *The Review*. This enables us to take a more optimistic view of the future, and we are certain that those undisciplined editorial tyros, Taft, White, Gorgas, Watt, *et al.*, will with us eagerly watch this Star in the West, until the journalistic standard is elevated away out of the reach of all the present editorial slow-coaches, who will have no resource save to profit by the extended experience and knowledge of the *Review* staff.

But, seriously, we cannot have too many good journals, and there is ability sufficient and a field broad enough in the West to make of the new one a shining success. It is to be entirely independent, and that is, to our mind, a good feature. It is projected by enthusiastic, earnest men, who really have something to say, and that is another. We sincerely hope that it will be adequately sustained, and that the editorial chair will be voluptuously cushioned, with ne'er a bent pin of captious criticism in it. We shall heartily welcome the new journal to an honorable place in dentistry.

